# Analysis of the Electroconsolidation Process of Fine-Dispersed Structures Out of Hot Pressed Al_2_O_3_–WC Nanopowders

**DOI:** 10.3390/ma14216503

**Published:** 2021-10-29

**Authors:** Edwin Gevorkyan, Mirosław Rucki, Zbigniew Krzysiak, Volodymyr Chishkala, Wojciech Zurowski, Wojciech Kucharczyk, Voskan Barsamyan, Volodymyr Nerubatskyi, Tomasz Mazur, Dmitrij Morozow, Zbigniew Siemiątkowski, Jacek Caban

**Affiliations:** 1 Wagon Engineering and Production Quality, Ukraine State University of Railway Transport, 7 Feuerbach Sq., 61010 Kharkiv, Ukraine; cermet-u@mail.com (E.G.); NeVlPa9@gmail.com (V.N.); 2 Faculty of Mechanical Engineering, Kazimierz Pulaski University of Technology and Humanities in Radom, Stasieckiego 54, 26-600 Radom, Poland; wojciech.zurowski@uthrad.pl (W.Z.); wojciech.kucharczyk@uthrad.pl (W.K.); tomasz.mazur@uthrad.pl (T.M.); d.morozow@uthrad.pl (D.M.); z.siemiatkowski@uthrad.pl (Z.S.); 3 Faculty of Production Engineering, University of Life Sciences in Lublin, Głęboka 28, 20-612 Lublin, Poland; 4 Department of Reactor Engineering Materials and Physical Technologies, V. N. Karazin Kharkiv National University, 4 Svobody Sq., 61022 Kharkiv, Ukraine; vchishkala@ukr.net; 5 Chair of Applied Physics, National Polytechnic University of Armenia, Vanadzor Branch, Vanadzor 2011, Armenia; barsamyan.voskan@mail.ru; 6 Faculty of Mechanical Engineering, Lublin University of Technology, Nadbystrzycka 36, 20-618 Lublin, Poland; j.caban@pollub.pl

**Keywords:** electroconsolidation, nanopowder, tungsten carbide, fast sintering, grain growth, diffusion

## Abstract

Fabrication of alumina–tungsten carbide nanocomposite was investigated. Characteristics of the densification and sintering were analyzed considering both the nano-size particle starting powders and the processing stages. Different heating rates were generated during densification and consolidation with a maximal load was applied only after a temperature of 1000 °C was reached. Due to the varying dominance of different physical processes affecting the grains, appropriate heating rates and pressure at different stages ensured that a structure with submicron grains was obtained. With directly applied alternating current, it was found that the proportion Al_2_O_3_ (50 wt.%)–WC provided the highest fracture toughness, and a sintering temperature above 1600 °C was found to be disadvantageous. High heating rates and a short sintering time enabled the process to be completed in 12 min, saving energy and time.

## 1. Introduction

Ceramic materials, including Al_2_O_3_, are very popular in industrial applications [1]. Alumina is characterized by high hardness and good strength, wear, and corrosion resistance, and it does not interact with metals during heating [2]. Despite the low fracture toughness of Al_2_O_3_ and the presence of strength-limiting flaws in the fabricated materials, aluminum oxide justifies evaluation because of many attractive properties [3]. The incorporation of refractory hard particles in Al_2_O_3_-based composites may inhibit grain growth in the material, significantly improving the mechanical properties of the composite [4]. Addition of WC accelerates the sintering process and helps in improving the mechanical properties of the Al_2_O_3_ matrix by hindering grain growth [5]. The authors obtained uniform microstructure with a grain size of 300–500 nm and demonstrated that the hardness and fracture toughness increased with decrease in alumina grain size. Experiments with other sintering technologies, such as hot pressing or vacuum compression sintering, showed that exposure to high temperatures leads to unfavorable grain growth which, in turn, worsens the mechanical properties of the sintered material [6]. Under spark plasma sintering (SPS), accelerated consolidation of powder materials can be performed at heating rates as high as 2500 °C/min [7]. The efficiency of coatings is greatly influenced by the application process itself and the specific parameters of technological spraying. Therefore, research related to the proper selection of spray parameters in order to ensure the resistance of the coating to various deterioration processes is a current topic of many scientific papers [8,9,10,11,12].

Qu et al. [13] reported results on two-step hot-pressing sintering (TSS) of WC (40 vol.%)–Al_2_O_3_ composites. Commercially available alumina powders consisting of amorphous Al_2_O_3_, boehmite (AlOOH), and χ-Al_2_O_3_, were used in the experiments, and the authors demonstrated the importance of both the pre-sintering stage aimed at obtaining critical initial density, and the isothermal stage of hot pressing, in which grain boundary migration played the main role. The achievement of a grain size of 2.38 µm at a relative density γ_rel_ = 99% was demonstrated, which provided an excellent hardness of 19.71 GPa, high fracture toughness of *K_IC_* = 12 MPa·m^1/2^, and flexural strength of 1285 MPa. The authors found that during the sintering process, the amorphous Al_2_O_3_, AlOOH and χ-Al_2_O_3_ were transformed completely to α-Al_2_O_3_. Dong et al. [14] used a hot-pressing sintering method to prepare a novel WC–Al_2_O_3_ whiskers composite with the addition of a VC-grain-growth inhibitor. The applied whiskers of hydrothermally synthesized ammonium aluminum carbonate hydroxide (AACH) transformed in situ into alumina and, due to retaining their morphology after sintering, contributed to an increase of fracture toughness up to 13.8 MPa·m^1/2^ and flexural strength up to 1335 ± 42 MPa, when the concentration of Al_2_O_3_ whiskers was 10 wt.%. However, a higher concentration of whiskers caused the formation of agglomerates and low-density regions, decreasing the strength of the composite.

The enhanced mechanical properties of WC-reinforced Al_2_O_3_ nano-composites can be obtained by modified spark plasma sintering (SPS) methods with starting powders of nanoscale particles. It is known that nanocrystalline powder consolidation, using various methods, enables full densification of nanopowders to be achieved and maintenance of nano size features, providing numerous benefits when lower sintering temperatures are applied, including elimination of the need for sintering aids [15]. However, the heterogeneous morphology of the plasmochemically synthesized oxide nanoscale particles contributes to the poor compressibility of the ultrafine powders [16]. A preliminary study [17] indicated the feasibility of a modified SPS method for nanopowder consolidation, and further studies of the formation of Al_2_O_3_–WC composite structures were then undertaken. Application of a modified field-assisted sintering technique enabled reduction of temperatures and sintering time below the most recently reported 1550 °C and 5 min [18]. This study demonstrated that lower temperature and shorter sintering time prevented grain growth, providing good mechanical properties with substantial energy and time savings. 

Our research addressed both theoretical and experimental issues. The main goal of the theoretical analysis was to identify dependence between surface effects in the contact zones of the sintered particles and process parameters, such as electric current, pressure, temperature, time, etc. The further impact on the mechanical properties of the sintered material needed to be demonstrated experimentally. The initial analysis provided the grounds for the assumption that surface diffusion was not the main sintering mechanism contributing to the formation of the nanostructured Al_2_O_3_–WC composite, which required differentiation of the heating rate during the process. Below we present the materials and methods, the theoretical background, and the results.

## 2. Materials and Methods

The experimental studies included:

- Microstructure analysis of Al_2_O_3_ and WC;
- Analysis of the dependence of densification on the sintering temperature: experiments were performed at 1000, 1100, 1200, 1300, and 1400 °C;
- Analysis of the dependence of densification on the heating rate: three heating rates were considered, namely, 50, 250, and 500 °C/min;
- Preparation of samples out of composite Al_2_O_3_ (50 wt.%)–WC using optimized sintering parameters, and analysis of their structure.

The investigations involved a process of powder consolidation under hot-pressing sintering conditions activated with an electrical field [19]. The device was described in [20], its only source of heat was the electricity applied to the graphite molds, so we found it appropriate to speak of ‘electroconsolidation’. Temperatures up to 1800 °C could be achieved, sufficient for the processes investigated. In the traditional electrical field-activated spark plasma sintering (SPS), the current is applied in the form of pulses [21], while the device used in our study utilized typical alternating current with a frequency of 50 Hz. It required only a simple transformer to obtain a high current of ca. 5000 A, with no additional units, such as pulse generators, needed. The electroconsolidation process took place in a vacuum chamber under a load able to provide a pressure of 45 MPa. The process creates a wide range of possibilities to obtain fine-dispersed high-density bulk materials out of both electrically conducting powders and non-conductors. 

Plasmochemically synthesized tungsten carbide (WC) powder of chemical purity 99.95% was used, with a particle size between 40 and 70 nm, made by Wolfram (Salzburg, Austria). Alumina powders supplied by Inframat (Manchester, CT, USA) were of similar grain size, between 60 and 80 nm. In order to obtain a homogenous blend, the powders were mixed in the planet ball mill “Pulverisette 6” (Fritsch GmbH, Idar-Oberstein, Germany), both dry and in isopropyl alcohol, over 2 h with a rotational speed of the disc 160 rev/min.

**Figure 1:**
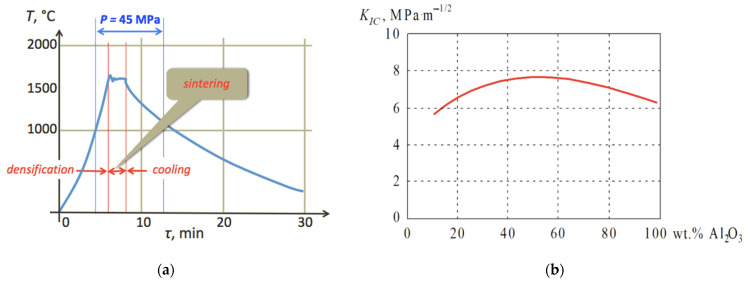
Experimental parameters: (**a**) Process of densification and sintering under electric current and pressure; (**b**) Effect of the composition on fracture toughness *K_IC_*.

The mixture of these powders was placed in graphite molds without any additional binders, and heated with different heating rates, 50, 250 and 500 °C/min. In the applied modified FAST/SPS (Field Activated Sintering Techniques/Spark Plasma Sintering) technology, compaction takes place when the powdered material is pressed and electric current is applied, heating it both directly and indirectly. When a temperature of 1100 °C was reached, the sample was loaded with pressure *P* = 45 MPa, but heating was continued up to 1600 °C. In this way the densification stage was completed, and the sample started to sinter. After 2 to 3 min in this temperature, the current was turned off, and the sample cooled down for some time under the load. The entire process lasted ca. 12 min, dependent on the heating rate at the beginning, allowing almost full densification and retaining small grains in the microstructure of the bulk material. A plot of the process stages is shown in Figure 1a, and the effect of the Al_2_O_3_ addition to the WC powder on the composite fracture toughness *K_IC_* is illustrated in Figure 1b. Based on these results, the research focused on the Al_2_O_3_ (50 wt.%)–WC proportion.

In the experiments presented in this paper, the typical heating rate was 250 °C/min, the sintering temperature was 1550 °C, and the holding time was 2 min.

Samples of diameter 12 mm and height 5 mm were prepared. In addition, 5 × 5 × 30 (base) mm samples were produced for the bending tests. The samples were investigated with the following methods: Bending strength was tested using the 3-point method with the machine MH-1 (Saratov, Russia), where the force was applied with 40 m/s speed. To determine each value, 5 samples were tested. Open porosity and density were measured by means of hydrostatic weighing. The error of the density measurement was below 1%. Hardness was measured with diamond pyramid indentation using a NEXUS 4504 microhardness tester (Innovatest, Maastricht, The Netherlands). From these measurements, both hardness and fracture toughness were calculated using the standard procedure [22]. The mass content of the components was determined according to the standard recommendations concerning alumina [23]. The mass percentage of the additions was determined with atomic emission spectroscopy (AES) [24]. Structural investigations involving starting powders, as well as fracture surfaces and grinded cross-sections of the sintered material, were performed using a JEOL JSM-840 scanning electron microscope (JEOL, Tokyo, Japan). In the X-ray structural analysis, XRD monochromatic CuK-α radiation was used of λ = 1.54187 Å using a Shimadzu XRD-6000 device (Shimadzu, Cambridge, UK). A constant scanning θ–2θ method was applied with scanning speed 1.2°/min, angle range 2θ = 5.0–100.0°, and step 0.02°; the X-ray tube had voltage 40 kV and current 30 mA; the sample was not rotated. Phase analysis was performed using the ASTM (American Society for Testing Materials) database.

## 3. Theoretical Background

Field-activated or current-assisted sintering techniques proved advantageous in sintering various hard to sinter materials, such as refractory, additive free, nano-structural or transparent ones [25]. In macroscopic models of sintering, it is convenient to treat strain rate $\dot{\varepsilon }$ as a composite of the respective elastic, thermal, and viscous components, $\dot{\varepsilon_{e}}$, $\dot{\varepsilon_{v}}$ and $\dot{\varepsilon_{T}}$ [26]. However, it was proved by Zeng et al. [27] that nanoscale sintering differs fundamentally from that of microscale in terms of kinetics and basic processes. The authors emphasized that in terms of the energy minimization principle, the driving force in nanoscale sintering was the reduction of the free surface energy. As a result, grain boundaries appeared temporarily and later disappeared due to mechanical rotation away from a low energy grain boundary orientation, which would not be expected according to standard microscale sintering theory [28].

During the heating process, physical contact between the particles develops in the network of the grain boundaries due to the free surface energy, while the excessive energy is the main driving force of the sintering process. The cleaner are the surfaces of the powder particles, the more uniform are the grains in terms of morphology. Under high heating rates, the sliding process along the boundaries is activated leading to quick densification of the sample. The sintering process is activated also due to lattice imperfections, which is substantial in the case of plasmochemically synthesized nanopowders.

**Figure 2:**
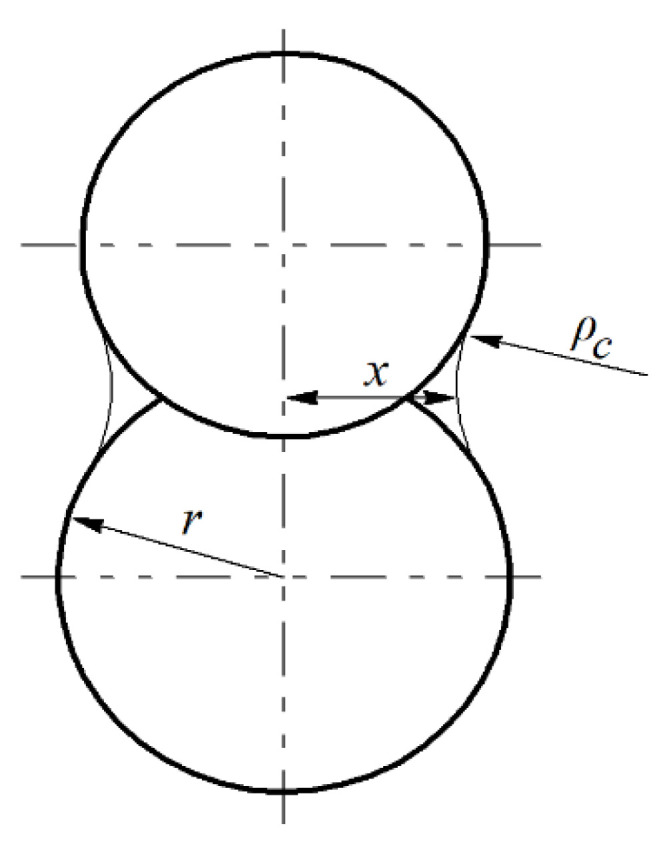
Contact area between the particles during densification.

Figure 2 illustrates increasing contact between the particles during the sintering process. According to Wakai et al. [29], the grain-boundary diffusion is driven by stress distribution or tension at the neck surface in the contact area. Thus, it is dependent on the dimensions of the particles and on compression at the center of the contact, as follows:

(1) $$\sigma =2\left(\gamma \kappa -\sigma_{n}\right)\left({\left[\frac{r_{c}}{x}\right]}^{2}-1\right)+\gamma \kappa ,$$

where *σ* denotes surface tension, *γ*—free surface energy, *κ*—curvature of the neck, *σ_n_*—average normal stress, *r_c_*—distance from the center of the contact, and *x*—radius of the contact area between particles.

Since the surface energy is larger than the boundary energy, there is a difference between the energies of vacancy appearance. Moreover, oversaturation of vacancies is different on the free surface of the neck and on the contact boundary between the particles. The sintering kinetics are dependent on gradient driven surface diffusion, as well as on grain boundary diffusion [27]. The kinetics of the contact surface growth between the particles depends exponentially on temperature and time according to:

*x*^ω^ = *A*(*T*) *τ*, (2)

where *A*(*T*) is a function of temperature *T*, particle geometry, and mass transfer mechanism, ω—exponent, *τ*—time. In the case of surface diffusion, the neck-growth exponent is ω = 7 [30], and the kinetic equation can be written as follows [21]:

*x*^7^ = 28*γ Ω D_S_ δ_S_ r*^3^*/RT τ*, (3)

where *Ω*—volume of voids, *D_S_*—surface diffusion coefficient, *δ_S_*—thickness of the surface diffusion layer, *r*—particle radius, *R*—gas constant.

The growth of boundaries takes place mainly as a result of surface diffusion. A methodology proposed by Johnson [31] enables description of densification considering diffusion in terms of volume, boundaries and surface. The respective coefficients can be obtained from experimental data. The mechanism of the viscous diffusion flow assumes that the boundaries between grains become channels for the voids without considering linear defects. In the case of nanoscale particles, dislocations are usually fixed to the surface. From the collected theoretical and experimental data, it is possible to predict the appearance and generation of dislocations during the sintering process. Under load, the atomic structure of the bulk material responds through deformation along the direction of the applied force [32]. In any sort of pressure-assisted sintering, the stress state during densification affects particle sliding and dimensional change due to the pore shrinkage [33]. The pressure difference between convex and concave particle surfaces induces shear which must exceed the threshold sliding stress *τ_s_* to generate particle rearrangement by sliding [34]:

*τ_s_* = 2*γ*/*r* + *γ*(1/*ρ_c_* − 1/*x*), (4)

where *ρ_c_* is the radius of the neck shown in Figure 2.

Various models of grain boundary sliding can be found in the literature [35]. Perhaps the earliest description of the grain boundary sliding in terms of diffusion-accommodated flow was provided by Ashby and Verral [36]. They assumed that the rate at which work was done by the applied stress, was equal to the power dissipated internally by diffusion, boundary sliding, interface reaction, and fluctuations. Under the simplification that boundaries act as perfect sinks and sources of vacancies, the grain-boundary tensile strain rate $\dot{\varepsilon_{g}}$ can be calculated as follows [36]:

(5) $${\dot{\epsilon }}_{g}=\frac{25\Omega }{RTr^{2}}\left(\gamma -\frac{0.72\delta_{S}}{2r}\right)D_{v}\left(1+\frac{3.3\delta_{S}D_{g}}{2rD_{v}}\right),$$

where *D_g_*—gas diffusion coefficient, *D_v_*—bulk diffusion coefficient.

Equation (5) is similar to the equation of the diffusional and viscous flow, but the deformation rate is of an order higher. Experimental data have confirmed the possibility of deformation caused by grain boundary sliding. Diffusion of the vacancies to the lattice and their interaction with dislocations form the flow of atoms to the contact zone between the particles. Moreover, interaction between vacancies and dislocations causes annihilation of the vacancies and creep of the dislocations. When the latter occurs parallel to the grain boundary, it causes grain displacement parallel to the boundary. The displacement value is then proportional to the overall dislocation line, creep distance parallel to the boundary, and to the Burgers vector divided by grain boundary area. The overall deformation is determined by the boundary sliding. The grain-boundary- and dislocation-mediated mechanisms can be competing or concurrent. The equation for the dislocation creep flow ${\dot{\varepsilon }}_{d}$ can be written as follows [36]:

(6) $$\varepsilon_{d}=A\frac{Gb}{kT}{\left(\frac{\sigma_{t}}{G}\right)}^{n}exp-\frac{E_{d}}{kT},$$

where *σ_t_*—uniaxial tensile stress, *G*—shear modulus, *b*—Burgers vector, *k*—Boltzman’s constant, *T*—temperature (°C), *E_d_*—activation energy for dislocation creep, *A* and *n*—experimental constants.

Since the grain boundary sliding mechanism described by Equation (5) is independent of the dislocation creep flow, Equation (6), the overall creep rate can be approximated by summing these two, as follows:

(7) $$\dot{\varepsilon }=\dot{\varepsilon_{g}}+\dot{\varepsilon_{d}},$$

Thus, it is reasonable to assume that in the low stress regime, activated sliding with diffusion accommodation prevails, while in the low stress regime, dislocation creep dominates. In the studied case of alumina nanopowders sintered with directly applied electric current, both mechanisms most probably occur, which provide near-theoretical density of bulk material at temperatures as low as 1100 °C. Such a temperature could be effective in the case of the sintering of pressed samples in a muffle furnace only when 1 wt.% Cu/Ti/Mg was added, providing uniform microstructure and relative densities up to 97% [37].

There are three main overlapping stages commonly distinguished in the sintering process, based on the connectivity of the solid and the porous phase [38]. In the initial stage, the bonding and necks between adjacent particles appear but densification is limited. The intermediate stage involves densification of the powder when the solid and the porous phase are connected. During the final stage, the pores are isolated and the solid phase is connected, and significant grain growth takes place in the crystalline materials, while interaction between pores and grain boundaries determines the microstructure of the sintered material. 

A high heating rate slows down the kinetics of the grain growth in the pure alumina. In the hot-pressing process activated with electric current, rearrangement of the particles takes place in the first stage, with dominant grain boundaries sliding and shear deforming contact areas of the particles and increasing the density up to 90%. In the intermediate stage, the role of the diffusion process increases, leading to the volumetric diffusion of all particles of the powder compact. Thus, densification during the hot-pressing process may be considered as material creep under pressure, determined by the grain boundaries sliding, as well as the volumetric and surface diffusion flow. The densification kinetics can be described by the Equation [7]:

(8) $$\dot{\theta }=\left(1-\theta \right)\left(\dot{\varepsilon_{x}}+\dot{\varepsilon_{y}}\right)=\left(1-\theta \right)\dot{\varepsilon_{x}},$$

where: *θ*—porosity, $\dot{\theta }$—densification rate (porosity change with time), $\dot{\varepsilon_{x}},\;\dot{\varepsilon_{y}}$—components of the shrinkage rate.

Densification kinetics can also be described using density and time, as follows:

(9) $$ln\left(\frac{1-\gamma }{1-\gamma_{0}}\right)=3\left(\frac{P}{\eta_{0}b}\right)ln\frac{1+b\tau }{4},$$

where: $\gamma_{0}$, γ—density at the point of initial pressure, and final density, respectively, *P*—specific pressure, $\eta_{0}$—initial viscosity, *b*—Burgers vector, *τ*—sintering time.

The theoretical equation of alumina nanopowder hot-pressing under electric current, based on the experimental data, can be written as follows:

(10) $$\frac{d\theta }{d\tau }=-\frac{3}{4}\frac{P}{\eta }\theta_{0}\tau ,$$

where: *θ_0_*—initial porosity of the powder compact.

Equation (10) demonstrates that the densification rate depends primarily on the applied pressure and viscosity of the material. Since the heating rate was very high, changes in viscosity and diffusion were not taken into consideration. When the electrically conductive powders are sintered with the current-assisted method, apart from high heating rate, the Joule heating contributes to the rapid densification. In contrast, non-conductive powders such as alumina undergo resistive heating from the graphite matrix and molds. The sintering time can be controlled by means of shrinkage measurement and is usually shorter than 10 min.

In the case of alumina nanopowders, unlike powders with particles larger than 100 nm, the particle surfaces are very active and developed. Alternating current prevents formation of agglomerates between the nano-size particles. Moreover, these powders are highly unstable thermodynamically with activation energies as low as 10–50 kJ/mol, due to polymorphic transformations during the heating. The grain growth kinetics of alumina largely depend on the specific area of the nanopowders, which is the main thermodynamic driving force of the sintering process. The sintering temperature of the nanopowders is 20–30% of the melting temperature, while in the case of micropowders, it can be 50 and even 80%. As a rule, the surface diffusion does not lead to the densification, i.e., neck form in nanoparticles does not depend on shrinkage, but it contributes to the grain growth. On the other hand, the surface diffusion is the mechanism most sensitive to grain growth and substantially affects it. In the case of nanopowders with large specific area and irregular particle shape, surface diffusion is helpful in low-temperature sintering. The experiments showed that, in the case of alumina nanopowders, it does not constitute the main sintering mechanism. Thus, it is reasonable to assume that the formation of nanostructured materials is not determined by the surface diffusion. Investigation of the necks between sintered nanoparticles provided evidence that the surface diffusion contributes to the dissolution of bulges on the nanoparticles providing clean surfaces.

In published reports, wide variation can be found in the calculated densification parameters of alumina, with activation energy ranging between 150 and 644 kJ/mol, so that no sole mechanism could be identified for the intermediate and final sintering stages [39]. In some cases, low activation energy and surface diffusion depend on the grain dimensions. Moreover, the creep process of grain boundary diffusion, viscous flow and, to a smaller degree, dislocation movement can be identified as factors determining the grain growth. Volumetric and surface diffusion resist the densification process, especially in the case of oxide nanopowders sintered at temperatures below those ensuring thermal stability. The clean surface of nanoparticles promotes minimization of sintering temperatures and thus formation of nanoscale structures in bulk material. A high heating rate limits grain growth, so that a rational densification rate can be obtained, in particular for α-Al_2_O_3_. In this system, a low heating rate promotes surface diffusion and related grain growth.

## 4. Results and Discussion

**Figure 3:**
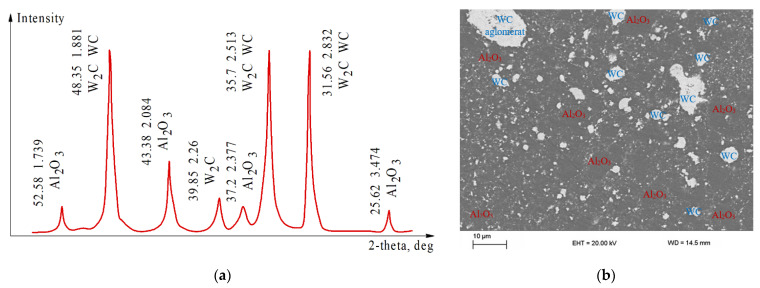
Analysis of the composite Al_2_O_3_ (50 wt.%)–WC, sintered at *T* = 1600 °C, *P* = 40 MPa, and holding time 2 min: (**a**) XRD; (**b**) micrograph of the grinded surface.

The sintered composites may have reduced durability due to the structural inhomogeneity that gives rise to generation of residual stresses during the manufacturing process [40]. From the XRD in Figure 3a, it can be seen in the specimen sintered at *T* = 1600 °C that some amount of W_2_C appeared as a process result. Many reports suggest that it is a common phenomenon in WC cement carbide systems for W_2_C and C to appear as a result of WC decomposition during thermal interaction [41]. As some reports have suggested [42], the presence of W_2_C might reduce the mechanical properties of the composite, even though its microstructure looks very advantageous in term of the dimensions and distribution of the grains. In Figure 3b, WC in the form of numerous sub-micron grains, as well as some larger agglomerates can be seen scattered in the alumina matrix.

**Figure 4:**
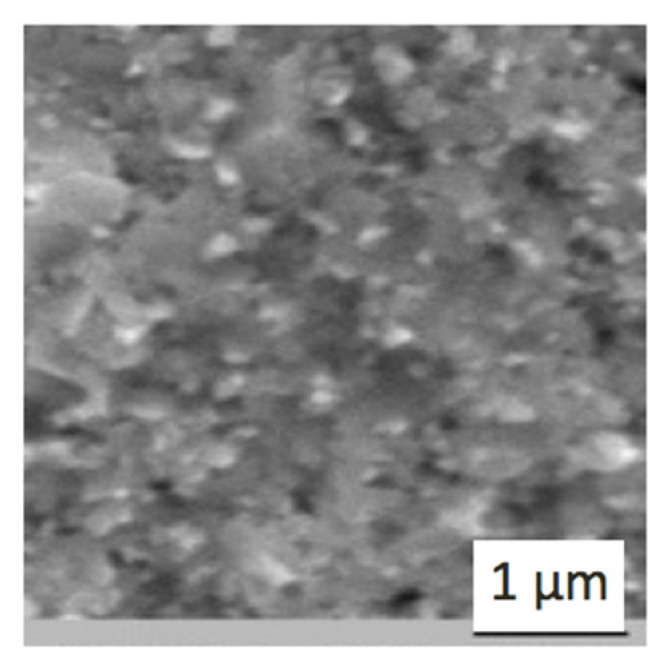
Microstructure of tungsten carbide obtained by electroconsolidation at *T* = 1500 °C, *P* = 45 MPa, holding time 2 min.

This result indicates that the high heating rate aimed at reduction of the surface diffusion may not be advantageous for some nanopowders. In some cases, grain boundary diffusion may play an important role in the densification process, especially in the case of nanopowders, more so than in case of micron-size particles. It may contribute to the higher activation energy, which promotes increase of the volume diffusion [43]. Therefore, alumina nanopowders are very sensitive to the heating rate, and the surface effects in the contact areas depend on the current, voltage and other electrical parameters. In our experiments, the local heating of the graphite mold contributed to the higher heating rate. The grain dimensions in the bulk material and density of the samples are shown in Table 1. An example of the sintered WC microstructure obtained at *T* = 1500 °C, under pressure *P* = 45 MPa, and with a holding time of 2 min, is shown in Figure 4.

In the experiments, the dimensions of grains in the sintered material remained submicron only at the heating rate of 500 °C/min, but they were ca. 5 times larger than the particles in the starting powders. This represents an improvement compared with a recent report [44] where two-step sintering was shown to be more effective in reducing the average grain size of micro α-alumina than existing sintering techniques. The authors obtained an average grain size of 0.94 µm applying a temperature of *T* = 1500 °C for 8 h, which is much more energy and time consuming than our experiments that provided a smaller grain size.

At the heating rates of 250 and 50 °C/min, the grain dimensions were, respectively, 30–40 and 60–90 times larger than the size of starting powders. It should be noted, however, that electroconsolidation at the lowest heating rate of 50 °C/min provided grain dimensions between 6 and 9 µm, while with the commercially available BO-13 cutting tool, and material obtained through the sintering method, Al_2_O_3_ grain size is ca. 2–5 µm [45].

From the perspective of densification, the porosity of bulk material is a crucial issue. In the experiments, the presence of larger and smaller pores was noted after electroconsolidation at any heating rate. It would be expected that the highest rate 500 °C/min could slow down the diffusional creep mechanisms contributing to the densification. However, it is not possible to identify all the mechanisms responsible for the densification even in traditional sintering processes. Analysis of the early stages of alumina sintering requires consideration of grain boundary diffusion [46], but sintering at high heating rates limits voids flow and decreases large pores. When the porosity at the grain boundaries is reduced, the mobility of boundaries increases. As a result, sintered nanopowders become sensitive to the heating, where two competing phenomena take place. On the one hand, large number of small pores appear, but on the other hand, these pores pose obstacles on the grain boundaries promoting grain growth. The dimensions of the alumina ceramic grains dependent on sintering time can be expressed by the following equation [47]:

(11) $$G=G_{0}+k\tau^{1/2},$$

where *G*_0_ and *G*—initial and final grain size, *k*—coefficient, *τ*—sintering time.

However, experimental data on the grain growth at different heating rates indicated that the proper equation for the grain dimensions that correspond with heating rate 500 °C/min can be written as follows:

(12) $$G=G_{0}+k\tau^{1/6},$$

**Figure 5:**
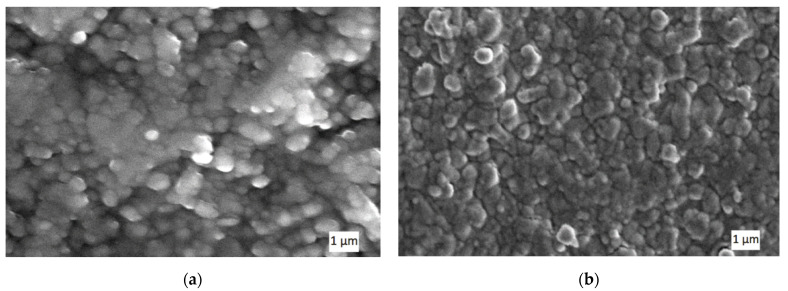
SEM images of Al_2_O_3_ sintered at heating rate 500 °C/min, *P* = 45 MPa, holding time 2 min, but different sintering temperatures: (**a**) *T* = 1500 °C; (**b**) *T* = 1600 °C.

The growth of grains in this case is slowed down by 66%, compared to that of conventional sintering without pressure. As a result, the grain size of alumina sintered at *T* = 1500 °C is similar to that obtained at *T* = 1600 °C, as can be seen in Figure 5.

**Figure 6:**
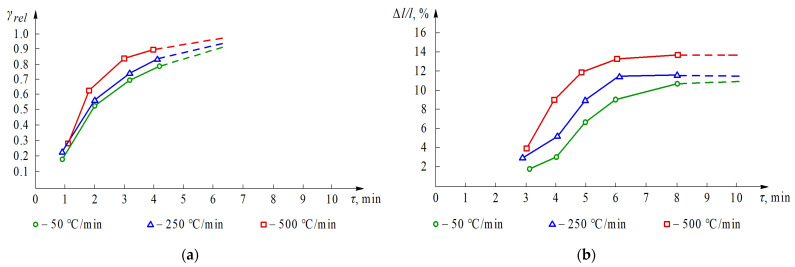
Effect of heating rate on; (**a**) relative density *γ_rel_*; and (**b**) shrinkage Δ*l*/*l*; of alumina nanopowders sintered at *T* = 1400 °C and *P* = 45 MPa.

Experimental research on the kinetics of the sintering process of alumina nanopowders has demonstrated that the densification rate depends on the temperature and pressure. Compared to the kinetics of WC nanopowders, Al_2_O_3_ is less dependent on the heating rate. Figure 6 presents the plots of relative density *γ_rel_* and shrinkage Δ*l*/*l* for alumina, and experimental results for different heating rates. Especially in terms of shrinkage (Figure 6b), between 3 and 6 min, the heating rate had an increased effect on the kinetics of densification. It should be noted that the relative density *γ_rel_* = 0.8 was reached after ca. 2.5 min of the process with a heating rate of 500 °C/min, but when the heating rate was 50 °C/min, it took more than 4 min. A high heating rate provided practically full densification in 6 min, so that almost no shrinkage was observed afterwards. Similar densification is unlikely to be obtained even after dozens of minutes at low heating rates.

**Figure 7:**
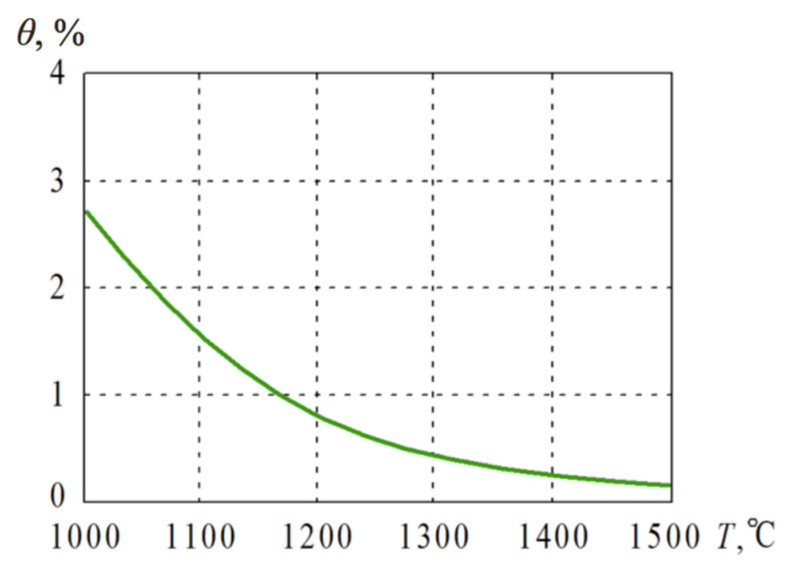
Porosity *θ* of the Al_2_O_3_ (50 wt.%)–WC ceramics obtained at different sintering temperatures.

When the electroconsolidation process was performed under pressure *P* = 45 MPa, the start and completion of the shrinkage were identified at 900 °C and 1600 °C, respectively. As a result, an increase of the sintering temperature provided higher density and respective mechanical properties. However, when the sintering temperature is higher than 1600 °C, it could be expected that no densification improvement takes place, while the grain growth might affect the structure and worsen the characteristics of the bulk material. Figure 7 presents the graph of porosity of the composite Al_2_O_3_ (50 wt.%)–WC obtained at different sintering temperatures. It is noteworthy that the percentage of pores did not decrease at temperatures higher than 1300 °C.

In order to confirm the optimality of the sintering temperature *T* = 1600 °C, additional samples were sintered at higher and lower temperatures and underwent analysis. The properties of the as-obtained specimens are shown in the Table 2.

The results in Table 2 demonstrate the worsening of all characteristics, especially of hardness and fracture toughness, which decreased by more than 10% at a sintering temperature *T* = 1650 °C. The density and bending strength decreased, too, but to a smaller degree.

Thus, it appears that the sintering temperature *T* = 1600 °C was optimal for the alumina–tungsten carbide composite sintered with the electroconsolidation method. Higher temperatures exceeded the temperature of phase interaction between WC and Al_2_O_3_, and led to the formation of CO, which, in turn, generated closed pores. The pressure of 45 MPa was limited by the graphite mold strength only, and future research will focus on the achievement of higher pressures. However, it is important to apply the maximal pressure only when the maximal temperature is reached to avoid the effect of the absorbed gases released from the powders afterwards [19].

## 5. Conclusions

The results of the study have informed conclusions concerning the heating rate and the microstructure of the obtained composites using the electroconsolidation method. Firstly, application of the starting powders with nanoscale particles made it possible to keep the grains of the sintered materials to sub-micron size, enhancing the mechanical properties of as-prepared composite. Both alumina and tungsten carbide exhibited better microstructures after sintering with high heating rates.

From a theoretical perspective, surface effects in the contact zones of the sintered particles depend on many factors that may be influenced with different parameters of electric current, pressure, temperature, and time. It was found that a sole heating rate properly applied was able to slow down the growth of grains by 66%, compared to that of conventional sintering. In the case of tungsten carbide, the high densification rate could be attributed to the Joule heating phenomenon between the conductive particles. However, at different stages of the sintering process, the high heating rate may affect the dominant densification and consolidation mechanisms, which required diversification of the heating process and related load application.

From a practical perspective, the proposed sintering method, activated by directly applied alternating current, is very successful for the fabrication of WC–Al_2_O_3_ composite ceramics with submicron grains. The Al_2_O_3_ (50 wt.%)–WC proportion gave the highest fracture toughness, and its optimal sintering temperature was 1600 °C at 45 MPa. To minimize porosity, the heating rate should be differentiated as follows: 50 °C/min before 500 °C is reached, then 250 °C/min up to 900 °C, and 500 °C/min up to 1600 °C. The maximal pressure should not be applied before at least 1000 °C is reached to avoid the formation of new pores out of absorbed gases. Further research will focus on strengthening the graphite mold to achieve higher pressures.

## Figures and Tables

**Table 1:** Density and Grain Dimensions in the Alumina and Tungsten Carbide Samples at Different Heating Rates.

Heating Rate, °C/min	Starting Density, g/cm^3^		Real Density, g/cm^3^		Grain Dimensions, µm	
	Al_2_O_3_	WC	Al_2_O_3_	WC	Al_2_O_3_	WC
50	0.52	0.9	3.95	15.3	6–9	2–3
250	0.51	0.7	3.93	15.5	3–4	0.5–1
500	0.52	0.7	3.95	15.7	0.5–0.6	0.5–0.75

**Table 2:** Properties of the Al_2_O_3_ (50 wt.%)–WC Ceramics Sintered at Different Temperatures.

No.	*P*, MPa	*T*, °C	*γ*, g/cm^3^	HV10	*σ_bend_*, MPa	*K_IC_*, MPa·m^1/2^
1	45.0	1500	3.95	15.3	50–60	6–8
2	45.0	1600	3.93	15.5	60–80	6–9
3	45.0	1650	3.95	15.7	60–80	5–6

## Data Availability

Data available on request due to privacy restrictions.
